# Multidetector computed tomography assessment of venous invasion in hepatic alveolar echinococcosis

**DOI:** 10.1007/s00261-022-03640-z

**Published:** 2022-10-07

**Authors:** Tieliang Zhang, Yuan Zhang, Jian Wang, Juan Hou, Wenya Liu

**Affiliations:** 1 Imaging Center, The First Affiliated Hospital of Xinjiang Medical University, Urumqi, 830011 Xinjiang China; 2 Imaging Center, The Fourth Affiliated Hospital of Xinjiang Medical University, Urumqi, 830011 Xinjiang China

**Keywords:** Hepatic alveolar echinococcosis, Multidetector computed tomography, Venous invasion, Diagnostic imaging

## Abstract

**Purpose:**

The objective of this study was to correlate multidetector computed tomography (MDCT) findings in hepatic alveolar echinococcosis (HAE) with intraoperative and postoperative histopathological results to identify reliable MDCT criteria for the diagnosis of HAE venous invasion.

**Methods:**

A total of 136 HAE patients who underwent CT examination were included in this study. The lesion-vessel contact angle, irregular wall, lumen stenosis and occlusion were evaluated.

**Results:**

A total of 614 veins were estimated. In total, 510 veins were invaded, and 104 veins were not. The invasion rate was 83.06%. In single CT findings, with a cutoff value of > 180° determined by receiver operating characteristic (ROC) curve analysis, the lesion-vessel contact angle performed the best (area under the ROC curve, AUC = 0.907, 95% confidence interval, 95% CI 0.872–0.941, *p* < 0.001), with a sensitivity, specificity and positive likelihood ratio (PLR) of 84.90%, 88.46%, and 7.35, respectively. Irregular wall and lumen stenosis showed the lowest diagnostic performance. Diagnostic performance was the highest when combining these criteria and signs (AUC = 0.932, 95% CI 0.905–0.960, *p* < 0.001).

**Conclusion:**

The lesion-vessel contact angle > 180° had the best sensitivity and specificity in the diagnosis of HAE venous invasion, and good interobserver agreement had been noted. The diagnostic performance of the lesion-vessel contact angle > 180° had been further improved with the addition of lumen occlusion accompanied by irregular wall or lumen stenosis.

**Graphical abstract:**

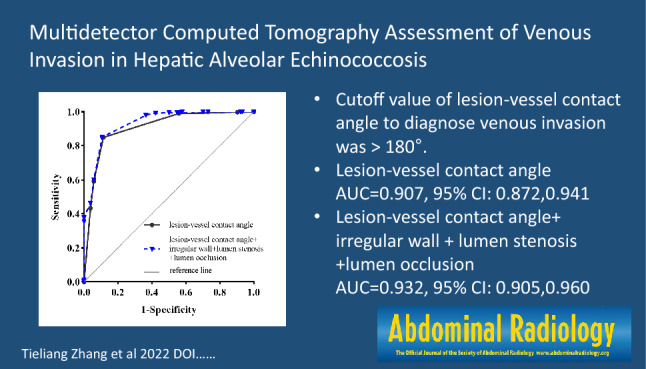

## Introduction

Echinococcosis is a parasitosis caused by cestodes of the genus Echinococcus and includes cystic echinococcosis (CE), alveolar echinococcosis (AE) and neotropical echinococcosis (NE) [1]. Echinococcosis has become a major global population health and economic problem [2, 3]. AE is endemic mostly in the northern hemisphere, including Europe, Asia and North America [4]. Almost all primary AE lesions occur in the liver [5]. Although hepatic alveolar echinococcosis (HAE) is considered to be a benign disease, the infection course is long and resembles that of liver cancer with characteristics of a tumor-like invasive growth pattern and the potential to develop metastases [6–9]. The mortality rate of untreated HAE patients within 10 years is as high as 90%, so it is also known as "insect cancer" [10–12].

Radical hepatectomy combined with antiparasitic agents is considered to be the best curative treatment option for HAE [8]. HAE has a long incubation period (5–15 years) and with an invasive growth pattern, therefore, HAE lesions are prone to invade multiple intrahepatic ducts, which makes routine resection particularly difficult [13, 14]. As a result, these patients can only receive albendazole anti-hydatid drug treatment, but long-term use of albendazole can cause a series of toxic and side effects [15]. Some studies suggest that a distance between the surgical margin and the edge of HAE lesion greater than 1 cm can remove the proliferative zone with infiltrating activity and achieve the standard of radical treatment [16, 17]. The condition of vascular invasion affects the choice of surgical operation methods, consequently, it is crucial to precisely evaluate the vascular invasion status within 1 cm of the HAE lesion edge, which will help clinicians to determine the lesion’s resectability, select rational surgical methods and design personalized treatment regimens. Accordingly, simple imaging diagnosis can no longer meet clinical needs, which makes it very important for radiologists to evaluate vascular invasion status.

At present, imaging studies on HAE mainly focus on metabolic activity, and FDG-positron emission tomography has been recognized as the preferred imaging method for detecting the metabolic activity of HAE lesions and surrounding areas [18–20]. Multi-energy/spectral CT, color and pulse Doppler ultrasound, and diffusion-weighted magnetic resonance imaging (MRI) may also be used in this regard [21–26], but few imaging studies focus on vascular invasion in HAE. Multidetector computed tomography (MDCT) has shown the best performance in HAE diagnosis, imaging classification and surgical treatment due to its excellent spatial resolution [26–28].

Previous studies have shown that the criteria or signs for MDCT diagnosis of tumor vascular invasion include lesion-vessel contact angle > 180°, lumen occlusion, lumen stenosis, and irregular wall [29–32]. Because surgeons are more concerned about venous invasion status and HAE is characteristic of vein involvement, we focused on the hepatic venous system in this study [33–35]. To date, there are no criteria that can be directly used to evaluate the venous invasion of HAE lesions. Consequently, the purpose of this study was to correlate the above criteria and signs with intraoperative and postoperative histopathological findings, determine the cutoff value for the diagnosis of HAE venous invasion and evaluate its accuracy to identify a reliable MDCT criterion for the diagnosis of HAE venous invasion.

## Methods and materials

### Study design and patient population

This retrospective study was conducted in accordance with the guidelines of the Declaration of Helsinki (revised in 2013). This study was approved by the Ethics Committee of the hospital (Approval No. 20190225-108, February 25, 2019), and the requirement for informed consent was waived. From January 2011 to December 2018, 165 patients admitted to our hospital with a clinical diagnosis of HAE were selected from the database of our hospital.

**Fig. 1:**
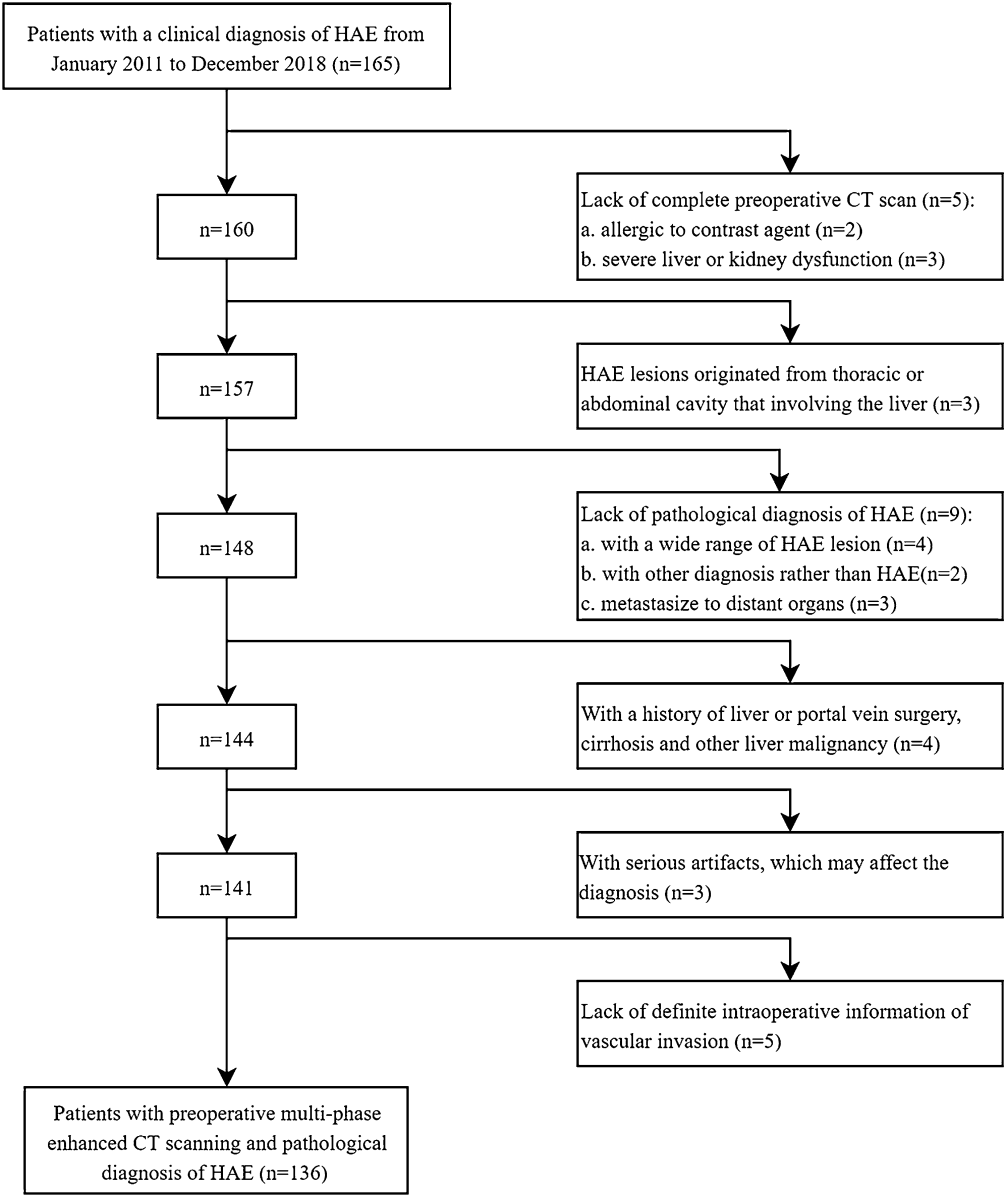
The flowchart of patient selection

The inclusion criteria were as follows: (1) the diagnosis of HAE was on the basis of clinical serological antibody testing or imaging examination, (2) a diagnosis of HAE was confirmed based on postoperative pathology, (3) the lesion originated from the liver rather than from the thoracic or abdominal cavity involving the liver, and (4) complete preoperative imaging data and surgical exploration records were available. Patients would be excluded if they had (1) a history of liver or portal vein surgery, cirrhosis and other liver malignancies, (2) allergies to contrast agents, (3) severe liver or kidney dysfunction, (4) images with serious artifacts, which may affect the diagnosis, and (5) incomplete medical records. The flowchart of patient selection was shown in Fig. 1.

### CT technique

The CT examination was performed with a multidetector spiral CT scanner (Discovery HD750, GE Healthcare, Milwaukee, WI, USA), and the patient in the supine position with arms raised. The scan ranged from the diaphragm to the lower margin of the liver. The scanning scheme included unenhanced scanning and multiphase enhanced scanning. The unenhanced scanning was followed by multiphase enhanced scanning, in which patients were injected with 1.5 mL/kg of body weight of nonionic iodinated contrast media (Iopromide, Ultravist 370, Schering, Berlin, Germany) at a rate of 3.5 to 4.0 mL/s through the antecubital vein by using a power injector (Tennessee-XD2003, Ulrich Medical, Ulm, Germany). Then, 20 mL of normal saline was injected at the same injection rate. The region of interest (ROI) was placed on the descending aorta at the diaphragmatic level. The triggering of the early arterial phase was monitored using an automatic tracking technique. When the CT value of the aorta reached 150 HU, the scan was automatically triggered. The arterial phase, portal venous phase and equilibrium phase were delayed by 25 s, 60 s, and 120 s, respectively. The scanning parameters were as following: tube voltage of 120 kVp with automatic tube current modulation, field of view (FOV) of 40 cm, slice thickness of 5 mm, slice interval of 5 mm, rotation time of 0.5 s, and reconstruction slice thickness of 1.25 mm.

### Image analysis

The original CT images were transferred to a postprocessing workstation (AW 4.4 Version, GE Healthcare, US). CT angiography (CTA) was performed by two experienced radiologists with more than 10 years of experience in the diagnosis of HAE. Reconstruction methods included volume reconstruction (VR), maximum density projection (MIP) and multiplanar reformation (MPR). The portal venous phase was used to evaluate all the veins, and the equilibrium phase served as a supplement. After reconstruction, all the images were transferred to the picture archiving and communication system (PACS), and the MDCT images were then reviewed by two radiologists who were blinded to the intraoperative findings and pathological reports. If any discrepancy was noted, the radiologists attempted to reach a consensus through discussion or consultation with a senior radiologist (with 25 years of abdominal imaging experience).

**Fig. 2:**
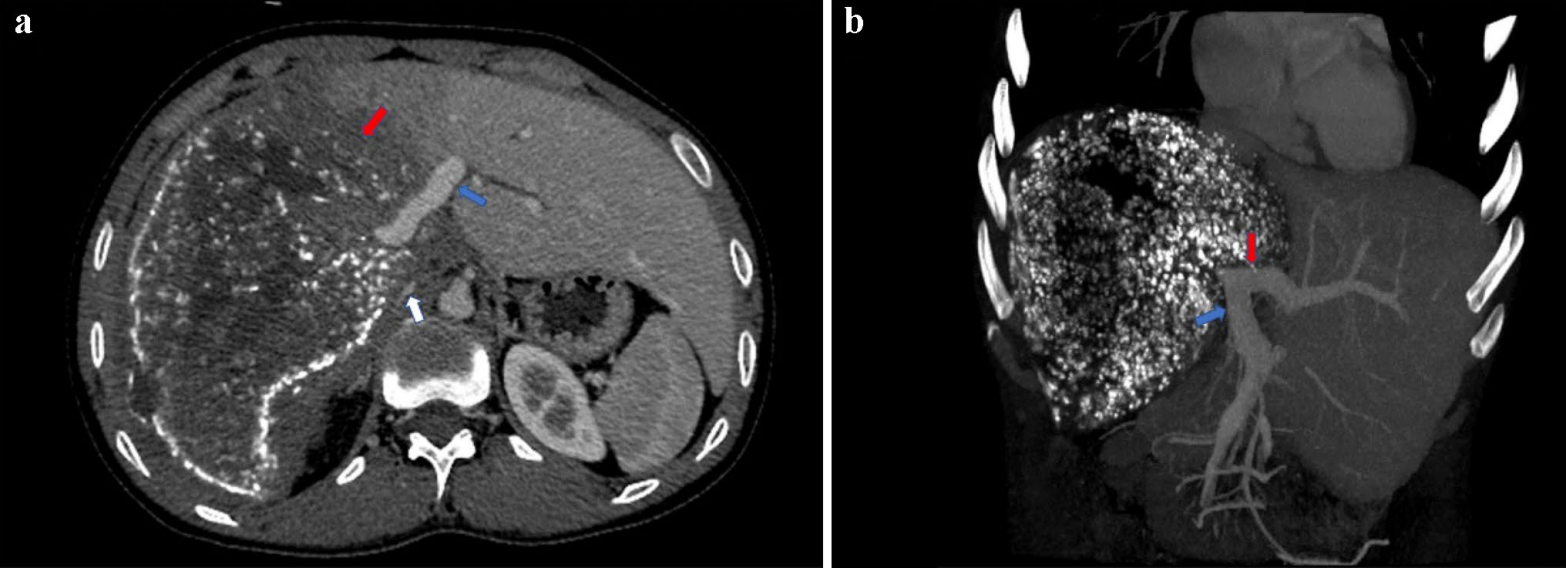
A 31-year-old female patient with HAE. **a** Axial CT image in the portal venous phase showed that the HAE lesion (red arrow) contacted the LPV (blue arrow), with the presence of an irregular wall and lumen stenosis. The RPV did not show due to lumen occlusion. The lesion-vessel contact angle of IVC (white arrow) was grade 5 accompanied by an irregular wall and lumen stenosis. **b** MIP image showed occlusion of the RPV, LPV (red arrow) showed with irregular wall and lumen stenosis, MPV (blue arrow) showed a little lumen stenosis. Pathological results confirmed that the LPV, RPV and IVC were all invaded by HAE, but MPV was normal. LPV, left portal vein; RPV, right portal vein; MPV, main portal vein; IVC, inferior vena cava; MIP, maximum density projection

**Fig. 3:**
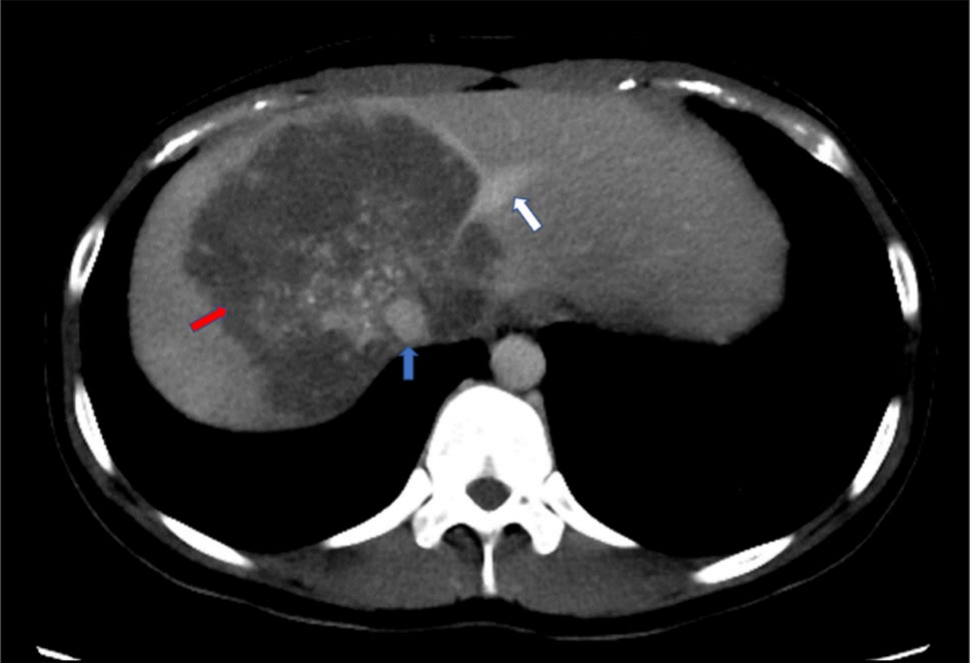
A 23-year-old female patient with HAE. Axial CT imaging in the portal venous phase showed HAE lesion (red arrow) had invaded the secondary porta hepatis, with absence of the RHV and MHV, the LHV (white arrow) was with lumen stenosis and irregular wall, and the lesion-vessel contact angle of IVC (blue arrow) was grade 4. Pathological examination confirmed the invasion of RHV, MHV, LHV and IVC. RHV, right hepatic vein; MHV, middle hepatic vein; LHV, left hepatic vein; IVC, inferior vena cava

In addition, we evaluated the portal vein (including the main portal vein, left portal vein and right portal vein), hepatic vein and inferior vena cava within 1 cm of the HAE lesion edge. The relationship between HAE lesions and veins was assessed by lesion-vessel contact angle, and other CT signs, including irregular wall, lumen stenosis and occlusion, were also evaluated (Figs. 2 and 3). Lu et al. [30] proposed a 5-grade system for tumor invasion of major vessels in pancreatic cancer patients. We modified Lu’s criterion from 5 to 6 grades. The details were as follows: grade 0, no contact between the lesion and vessel; grade 1, greater than 0° and equal to or less than 90°; grade 2, greater than 90° and equal to or less than 180°; grade 3, greater than 180° and equal to or less than 270°; grade 4, greater than 270° and less than 360°; and grade 5, equal to 360°. On the postprocessing workstation, we assessed the lesion-vessel contact angle on the short-axis image of the invaded vein.

This study had a definite gold standard (intraoperative findings and pathological reports) for venous invasion. Venous invasion was defined as infiltration of the vein by HAE lesion and cannot completely be dissected by the surgeon during intraoperation; on the other hand, we can also evaluate venous invasion from final pathological reports.

### Statistical analysis

SPSS 26.0 software (IBM, Armonk, New York, USA) was used for statistical analysis. The data in this study were described by descriptive statistics. Mean and standard deviation were used to describe variables that conformed to normal distribution, and median and interquartile range were used for variables that exhibited a nonnormal distribution. The sensitivity, specificity, positive predictive value (PPV), negative predictive value (NPV), accuracy, positive likelihood ratio (PLR) and negative likelihood ratio (NLR) were calculated to evaluate the diagnostic efficiency for venous invasion of different findings. The cutoff value of the lesion-vessel contact angle and its diagnostic performance in combination with other signs were calculated by receiver operating characteristic (ROC) curve analysis. Variation between two observers was assessed by Cohen's Kappa test. Here, *p* < 0.05 was considered statistically significant.

## Results

There were 136 patients in this study. A total of 614 veins were estimated, including 237 portal veins, 272 hepatic veins, and 105 inferior vena cava. Veins with lesion-vessel contact angle > 180° accounted for 71.98% of all veins including in this study. Among the 614 veins, 510 veins were invaded, and 104 veins were not, with an invasion rate of 83.06%. Among the invaded veins, there were 22 main portal veins (MPV), 84 right portal veins (RPV), and 72 left portal veins (LPV). There were 240 hepatic veins and 92 inferior vena cava. Demographic and baseline data were shown in Table 1.

**Table 1:** Demographic and baseline data

Variables	Findings
Age, years	38.08 ± 13.06
*Gender*	
Male	65/136 (47.80%)
Female	71/136 (52.20%)
*Ethnicity*	
Tibetan	57/136 (41.90%)
Kazakh	32/136 (23.50%)
Han	25/136 (18.40%)
Other	22/136 (16.20%)
*Location*	
Right lobe	59/136 (43.40%)
Left lobe	23/136 (16.90%)
Right and left lobe	54/136 (39.70%)
Maximum dimension (cm)	13.46 ± 4.27
*Lesion-vessel contact angle*	
Grade 0	11/614 (1.79%)
Grade 1	39/614 (6.35%)
Grade 2	122/614 (19.87%)
Grade 3	137/614 (22.31%)
Grade 4	81/614 (13.19%)
Grade 5	224/614 (36.49%)
BMI (kg/m^2^)	22.30 ± 3.73
RBC (× 10^9^/L)	7.35 (5.90, 9.02)
WBC (× 10^9^/L)	4.44 ± 0.67
HGB (g/L)	129.50 (113.22, 139.75)
PLT (× 10^9^/L)	279.50 (221.00, 338.25)
A/G	0.89 (0.62, 1.12)
AST (IU/L)	26.45 (20.50, 55.65)
ALT (IU/L)	30.90 (17.13, 63.48)
PT (S)	12.30 (11.52, 13.20)
TT (S)	20.78 ± 1.74

*BMI* body mass index, *WBC* white blood cell, *RBC* red blood cells, *HGB* hemoglobin, *PLT* platelet, *A/G* albumin-globulin ratio, *AST* aspartate transaminase, *ALT* alanine transaminase, *PT* prothrombin time, *TT* thrombin time

Grade 0, no contact between the lesion and vessel; Grade 1, greater than 0° and equal to or less than 90°; Grade 2, greater than 90° and equal to or less than 180°; Grade 3, greater than 180° and equal to or less than 270°; Grade 4, greater than 270° and less than 360°; and Grade 5, equal to 360°

Evaluating interobserver consistency, the Cohen’s kappa value for the lesion-vessel contact angle was 0.878, and the Cohen’s kappa values for the presence or absence of irregular wall and lumen stenosis were 0.652 and 0.796, respectively. The Cohen’s kappa was 0.945 for the presence or absence of lumen occlusion. Discussions should be held for poor interobserver consistency (Cohen kappa < 0.75).

The estimated optimal cutoff value of lesion-vessel contact angle for the diagnosis of venous invasion was > 180°. Using a cutoff value of > 180°, the lesion-vessel contact angle showed a sensitivity of 84.90%, a specificity of 88.46%, and a PLR of 7.35 in evaluating venous invasion by HAE, the area under the ROC curve (AUC) = 0.907 (95% confidence interval [95% CI] 0.872–0.941, *p* < 0.001).

For evaluating the venous invasion situation, the lesion-vessel contact angle showed the best diagnostic performance compared with irregular wall, lumen stenosis and lumen occlusion. However, irregular wall and lumen stenosis showed low sensitivity and specificity. Interestingly, lumen occlusion showed the best specificity of 100.00% but with the lowest sensitivity (Table 2).

**Table 2:** Diagnostic performance of CT criterion and signs

	SEN	SPE	ACC	PPV	NPV	PLR	NLR
Irregular wall	61.17%	51.92%	59.60%	86.18%	21.42%	1.27	0.74
Lumen stenosis	62.74%	43.26%	59.44%	84.43%	19.14%	1.10	0.86
Lumen occlusion	38.03%	100.00%	48.53%	100.00%	24.76%	N/A	0.61
Lesion-vessel contact angle	84.90%	88.46%	85.50%	97.30%	54.43%	7.35	0.17

*SEN* Sensitivity, *SPE* Specificity, *ACC* Accuracy, *PPV* positive predictive value, *NPV* negative predictive value, *PLR* positive likelihood ratio, *NLR* negative likelihood ratio, *N/A* not applicable

**Fig. 4:**
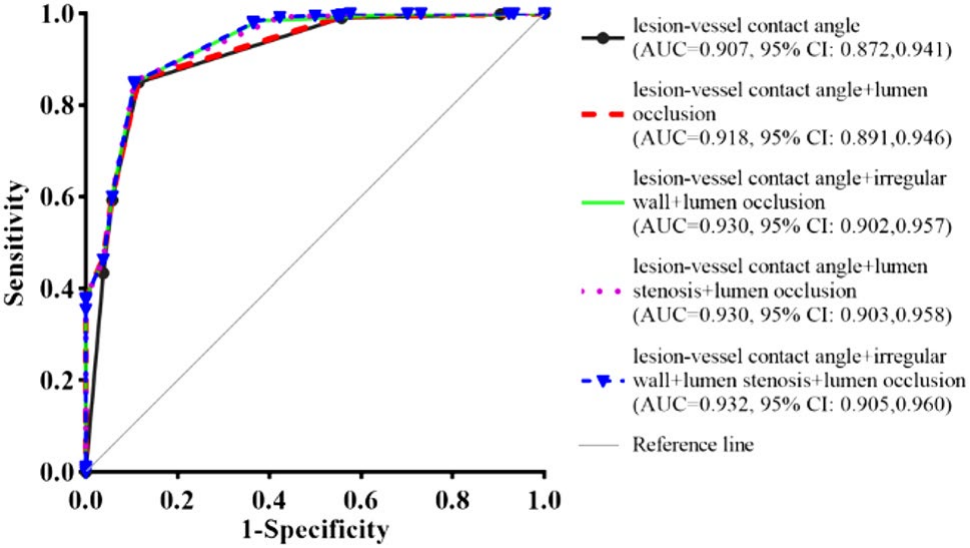
Receiver operating characteristic curves of the lesion-vessel contact angle and combined with other CT signs

In single CT findings, with a cutoff value of > 180°, the lesion-vessel contact angle showed the best diagnostic performance compared with other CT signs. Lumen occlusion showed the highest specificity, but with the lowest sensitivity, combining with lesion-vessel contact angle > 180° can also improve diagnostic performance. Irregular wall and lumen stenosis showed the lowest diagnostic performance, even when they were combined with lesion-vessel contact angle > 180° alone or in combination, the performance was not better than the latter. Only when irregular wall and lumen stenosis were associated with lumen occlusion, the diagnostic performance was better than lesion-vessel contact angle itself. Diagnostic performance was the greatest when all the criteria and signs were present simultaneously (AUC = 0.932, 95% CI 0.905–0.960, *p* < 0.001) (Fig. 4).

## Discussion

This study included 136 patients. The mean age was 38.08 ± 13.06 years, and the mean maximum dimension was 13.46 ± 4.27 cm. These results were different from Europe centers in patient age and maximum dimension of the lesion, Chinese patients were younger, and the lesion size were larger than European patients [36]. Possible reasons maybe the more frequent contact that children in China may have with infected dogs because of a pastoral lifestyle, higher environmental infection pressure. On the other hand, because of residence in remote rural areas, Chinese HAE patients commonly do not seek medical treatment until they are symptomatic, so HAE patients in China are diagnosed late and the lesion sizes are quite large. A total of 614 veins were estimated with a high invasion rate of 83.06%. In this study, we assessed veins within 1 cm of the HAE lesion edge, those veins more than 1 cm were not included in this study. Therefore, the invasion rate of this study was different from the study of vascular invasion based on MRI examination, in which the invasion rate of portal veins and hepatic veins were 51.88%, 43.28%, respectively [34].

Due to its high-density resolution and high scanning speed, MDCT has shown great advantages in the preoperative diagnosis of HAE, morphological classification, preoperative patient evaluation, PNM staging evaluation, postoperative follow-up, and determination of an appropriate treatment method. At present, there are several studies on CT vascular invasion, which mainly focus on pancreatic cancer and hilar cholangiocarcinoma [29–31, 37–40]. However, there has long been a lack of recognized CT criteria for the assessment of HAE vascular invasion. Although HAE and tumors have similar biological characteristics, the criteria for vascular invasion in pancreatic cancer or perihilar cholangiocarcinoma cannot be directly applied to HAE.

Using the ROC curve, we found that the cutoff value of the lesion-vessel contact angle for venous invasion was > 180° and the diagnostic efficiency was good, the sensitivity, specificity were 84.90%, 88.46%, respectively. Lu's criteria for diagnosing pancreatic cancer, with lesion-vessel contact angle > 180° as the threshold, the sensitivity was similar with ours, while the specificity was 98%, which was higher than ours, the possible reasons were different prevalence and characteristics of the two lesions [30]. The cutoff value was the same as Lu's criterion and NCCN's criterion for vascular invasion of pancreatic cancer. The NCCN guideline indicated that when the tumor was in contact with the SMV or PV of > 180°, the tumor was considered unresectable [41]. Although the PPV was as high as 97.30%, NPV was very low. The prevalence can affect both PPV and NPV. Usually, the higher the prevalence, the higher the PPV. PLR is not affected by the prevalence. In this study, the probability of the lesion-vessel contact angle > 180 in the invaded veins was 7.35 times higher than that in the non-invaded veins.

In single CT criterion and sign, the lesion-vessel contact angle with a cutoff value of > 180° performed better than other signs. The diagnostic performance of irregular wall and lumen stenosis were the lowest with low sensitivity and specificity. The possible reason is that the venous wall is thinner than the arterial wall, so lumen stenosis and irregular wall are easily observed when HAE lesion present. Even if lumen stenosis and irregular wall were assessed alone or combination with the lesion-vessel contact angle, the difference was not statistically significant compared with the latter. The possible reasons may be the lack of a systematic evaluation of these two grading standards, which were susceptible to the influence of observer experience. Interestingly, lumen occlusion had a good specificity of 100% but a very low sensitivity of 38.03%. In this study, there were 194 veins with lumen occlusion, which were confirmed by intraoperative exploration and postoperative pathology. The venous lumen is larger, and the blood stream is typically slow. HAE lesion can easily infiltrate into the lumen and form embolus, causing lumen occlusion gradually.

Although the diagnostic performance of the lesion-vessel contact angle > 180° was better than other CT signs, it was also associated with false-positive and false-negative findings. There were 89 veins that had been wrongly judged, so there was an urgent need to improve its diagnostic performance. When the lesion-vessel contact angle was > 180°, the diagnostic performance was significantly improved when accompanied by occlusion, and the difference was statistically significant. When the lesion-vessel contact angle was > 180° with irregular wall or lumen stenosis, the diagnostic efficiency can be significantly improved only when this metric was accompanied by occlusion. When combining these criteria and signs, the diagnostic efficiency was the highest.

In this study, we assessed the diagnostic performance of the CT criteria and signs for HAE venous invasion including lesion-vessel contact angle > 180°, irregular wall, lumen stenosis and occlusion. We found that the lesion-vessel contact angle > 180° was the most useful as a diagnostic criterion for HAE venous invasion. When combining these criteria and signs, the diagnostic performance reached the highest. These findings may aid decision-making for radiologists and clinicians regarding the assessment of venous invasion in HAE patients. Up to now, the only CT study on HAE vascular invasion in China had no clear criteria for vascular invasion, and vascular invasion was classified into three grades based on imaging findings [42]. In this study, the lesion-vessel contact angle was a semi-quantitative criterion, which can more accurately assess venous invasion status compared with other CT signs.

Vascular invasion criteria of HAE by MRI were as follows: incomplete low vascular wall signal in T1 weighted image (T1WI), vascular truncation, surrounding by lesion, lumen stenosis or occlusion. However, lumen stenosis but with a completely low vascular wall signal on T1WI was not considered vascular invasion [30]. Some of these criteria were similar to ours. However, CT examination could not accurately evaluate the low vascular wall signal, so we described the vascular wall contour and the presence or absence of an irregular wall. In this study, the diagnostic performance of lumen stenosis was poor with a specificity of 43.26%, and 59 vessels with lumen stenosis were incorrectly diagnosed as venous invasion. As we all know, in addition to the vein, HAE can also invade the artery. According to a study based on MRI, the incidence of arterial invasion by HAE lesion was 26.87% [34]. We focused on veins in this study, the MDCT criteria for evaluating venous invasion included lesion-vessel contact angle > 180°, irregular wall, lumen stenosis and occlusion. Whether these criteria can be applied directly to the artery need further confirmation. Several studies have shown that arterial invasion and venous invasion should be treated separately due to different imaging signs. Veins are more susceptible to invasion mainly because the venous wall is thinner and the venous lumen is larger than the arteries [43].

There were several limitations in this study. First, this was a single-center study, and potential selection bias may have existed. Second, each vein may have different anatomical trends or properties, and this study did not assess the portal vein, hepatic vein and inferior vena cava separately. Third, in this study, due to the characteristics of HAE invading veins, the hepatic arteries were not included in the study. However, in clinical work, some hepatic arteries will also be affected. Finally, the criteria and CT signs were subjective and varied from observer to observer, and some potential features of HAE lesions could not be recognized by the naked eye. Computer-aided diagnostic systems are expected to solve this disadvantage in the future. In subsequent studies, we will combine with other centers, include hepatic arteries and classify the veins to explore differences in imaging signs of arterial and venous invasion. In addition, artificial intelligence will be used to study vascular invasion in order to show its internal characteristics. Although there are many limitations or deficiencies in this study, the observed discrepancies between imaging and intraoperative findings or pathological reports have major impact on clinical decision making and selection of appropriate treatment strategies.

## Conclusion

A lesion-vessel contact angle > 180° had the best sensitivity and specificity in the diagnosis of HAE venous invasion, and good interobserver agreement had been noted. The diagnostic performance of the lesion-vessel contact angle > 180° had been further improved with the addition of lumen occlusion accompanied by irregular wall or lumen stenosis.
